# The Role of Gut Microbiota and Diet on Uremic Retention Solutes Production in the Context of Chronic Kidney Disease

**DOI:** 10.3390/toxins10040155

**Published:** 2018-04-13

**Authors:** Laetitia Koppe, Denis Fouque, Christophe O. Soulage

**Affiliations:** 1 Department Nephrology, Centre Hospitalier Lyon Sud, F-69495 Pierre-Benite, France; denis.fouque@chu-lyon.fr; 2 CarMeN Lab, INSA-Lyon, INSERM U1060, INRA, University Lyon 1, F-69621 Villeurbanne, France; christophe.soulage@insa-lyon.fr

**Keywords:** chronic kidney disease, intestinal microbiota, pro/prebiotics, vegetarian diet, low protein diet, nutrient composition, uremic toxins

## Abstract

Uremic retention solutes (URS) are associated with cardiovascular complications and poor survival in chronic kidney disease. The better understanding of the origin of a certain number of these toxins enabled the development of new strategies to reduce their production. URS can be classified according to their origins (i.e., host, microbial, or exogenous). The discovery of the fundamental role that the intestinal microbiota plays in the production of many URS has reinstated nutrition at the heart of therapeutics to prevent the accumulation of URS and their deleterious effects. The intestinal microbiota is personalized and is strongly influenced by dietary habits, such as the quantity and the quality of dietary protein and fibers. Herein, this review out lines the role of intestinal microbiota on URS production and the recent discoveries on the effect of diet composition on the microbial balance in the host with a focus on the effect on URS production.

## 1. Introduction

Patients with chronic kidney disease (CKD) exhibit a high risk of cardiovascular (CV) diseases due to accelerated atherosclerosis and metabolic complications like dyslipidemia and perturbation of glucose homeostasis. Beside traditional CV risk factors present in the vast majority of CKD patients, specific CV risk factors for CKD have been established in the literature, such as oxidative stress and inflammation [1]. In particular, compounds retained by the failing kidney called uremic retention solutes (URS) have emerged as another potential CV and metabolic risk factors for patients with CKD.

The human gastrointestinal tract (GIT) contains trillions of microorganisms, collectively referred to as the gut microbiota [2]. Gut microbiota establishes a symbiotic association with its host and is involved in the regulation of multiple host metabolic pathways, giving rise to interactive host–microbiota metabolic, signaling, and immune-inflammatory axes that physiologically connect the gut to liver, muscle, adipose tissue, and even brain. The microbiota also contributes to metabolism by producing nutrients (e.g., short-chain fatty acids, SCFAs), energy from the diet, and vitamins (e.g., vitamin K) [3]. 

In contrast, it has been reported that gut microbiota can also produce various potentially harmful metabolites. Uremia results in an alteration of the composition of the gut microbiota often referred to as “uremic dysbiosis” [4]. Dysbiosis is defined by an impaired balance between symbionts (normally harmless) and pathobionts (which exert pathogenic effects on the host) in a way that favors pathobiont overgrowth. Uremic dysbiosis may be due to iatrogenic causes (e.g., antibiotics, phosphates binders, antimetabolites, …) or uremic environment per se. The imbalance of intestinal microbiota and deleterious colonic microbial metabolism affects the production of microbiota-derived metabolites. Thus, the composition and balance of the gut microbiota could strongly contribute to the accumulation of URS observed in CKD. 

Dialysis is able to remove a substantial proportion of URS. However, this technique is only implemented in patients with end-stage renal disease. Also, among the three physicochemical types of URS [5], the pathophysiologic importance of protein-bound toxins has been found to be important in CV complications. During dialyses, only the free fraction of protein-bound solutes can diffuse across the dialysis membrane, resulting in very limited removal. It is therefore urgent to develop new strategies (other than dialysis techniques) to reduce plasma concentrations of major URS from early stages of CKD. Indeed, an alternative means to lower plasma URS levels is to reduce their production. The gut microbiota is characterized by an inter-individual variability due to genetic and environmental factors and disease state. Among the environmental ones, dietary habits play a key role in the modulation of gut microbiota composition and could be an attractive strategy to modulate URS production in CKD and limit their deleterious effects on the tissues. Such a strategy could also benefit a much larger population of CKD patients before the initiation of renal suppletion therapy. 

For the purpose of this review, we will define the potential origin of major URS and their respective toxicity and will then focus on recent nutritional strategies, such as vegetarian diet, pre/probiotics, and low protein diet (LPD), used as tools to decrease URS accumulation in the context of CKD. 

## 2. Origins of URS and Clinical Consequence of the Accumulation of Intestinal URS 

The major interest in URS during uremia led to the creation of the European Uremic toxin work group (EUTox, www.uremic-toxins.org) involved in the identification, classification, and characterization of URS [5]. The “operational” classification of the URS by EUTox was originally based on the main physico-chemical characteristics that dictate their behaviors during dialysis processes. The classification of the uremic toxins was therefore mainly based on their size and their protein binding properties, leading to 3 classes of compounds: (1) the low molecular weight (<500 Da) water-soluble compounds; (2) the middle molecules (500–60,000 Da) and (3) the low molecular weight protein-bound compounds. Over the past decade, many studies have shed light on the production mechanisms and today an alternative classification of URS could be proposed according to their origin: 1-endogenous metabolism; 2-microbial metabolism, or 3-exogenous intake. 

The majority of gut-derived uremic toxins have shown a direct role in mortality and complications associated with CKD. For example, indoxyl-sulfate (IS) and p-cresyl-sulfate (PCS) are positively correlated with an increased mortality in CKD patients [6]. Trimethylamine N-oxide (TMAO) was recognized as a pro-atherogenic metabolite [7] and was associated with CV disease in the general population [8] as well as in patients with CKD [9,10].

Dimethylglycine (DMG) has also been associated with an increased risk of CVD [11]. However, the precise dysmetabolism leading to the accumulation of DMG in CKD is still questionable. In the liver and kidney, choline is oxidized into betaine. This is a two-step enzymatic reaction in which choline is first converted to betaine aldehyde, a reaction catalyzed by the mitochondrial choline oxidase and betaine aldehyde is further oxidized in the mitochondria or cell cytoplasm to betaine by betaine aldehyde dehydrogenase. Finally, betaine is converted into DMG by betaine-homocysteine methyl transferase (BHMT). Some data highlighted that beside intestinal dysbiosis, an increase of BHMT activity could also contribute to CV disease. Betaine acts as a methyl donor in the conversion of homocysteine to methionine in the presence of BHMT. DMG accumulates in CKD and is an independent predictor of plasma homocysteine concentrations, a well-known risk factor for atherosclerosis [12]. Glutarate is described as a neutotoxic metabolite as observed in glutaric acidemia type I, a neurometabolic disease caused by the deficiency of glutaryl-CoA dehydrogenase, which leads to the tissue accumulation of glutarate [13]. However, no data are available about its potential toxicity in CKD patients. 

The view that urea is simply an innocent bystander was challenged by several recent publications [14]. Firstly, ammonia derived from urea has been reported to disrupt the intestinal epithelial barrier, which is involved in the pathogenesis of inflammation in CKD [15]. Secondly, urea could directly perturb glucose homeostasis by promoting insulin resistance [16] and beta-cell dysfunction [14], two hallmarks of type 2 diabetes. Thirdly, urea can contribute to post-translational modification of proteins via the breakdown product cyanate through a process called protein carbamylation which promotes atherosclerosis and mortality in CKD [17].

## 3. Role of Intestinal Microbiota on URS Production 

**Figure 1:**
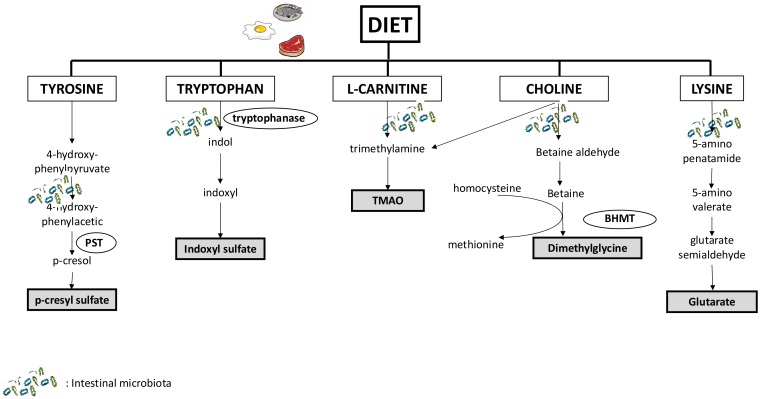
Major pathways involved in the production of uremic retention solutes from nutrients and the interplay with intestinal microbiota. Abbreviation: TMAO: trimethylamine N-oxide; PST: phenolsulfotransferase; BHMT: betaine-homocysteine methyl transferase.

The role of the intestinal microbiota in the production of many URS has been unambiguously demonstrated (Figure 1). Since the pioneering work by Einheber and Carter [18] who reported that in rats with bilateral nephrectomy, the absence of microbiome prolonged life expectancy, there are many experimental and clinical study in human and animal to demonstrate the pivotal role of gut in CKD [4,19,20]. Because numerous metabolites accumulate during CKD, it is difficult to accurately define the systemic contribution of the microbiota in the accumulation of URS and the actual impact of gut microbiota on the accumulation of URS remains not fully understood. Recent data, using germ free mice (the so-called axenic mice), have helped to characterize the origin of URS and target those that will be most sensitive to nutritional strategies [21,22]. In particular, using a spectrometry-based approach in these models, 11 URS were identified as being microbiota-derived uremic solutes, including indoxyl sulfate (IS), p-cresyl sulfate (PCS), phenyl sulfate, cholate, hippurate, dimethylglycine (DMG), γ-guanidinobutyrate, glutarate, 2-hydroxypentanoate, trimethylamine N-oxide (TMAO), and phenaceturate.

Metabolome profiling showed that these solutes could be classified into three groups depending on their origins: (1) Compounds entirely derived from gut microbiota (e.g., PCS and IS). Indeed, the gut microbial fermentation of dietary tryptophan and tyrosine is the unique source of IS and PCS, respectively; (2) Compounds derived from both host and gut microbiota (e.g., DMG and glutarate). Some anaerobic bacteria are involved in the production of betaine aldehyde and 5-aminovalerate, which are the precursors of dimethylglycine and glutarate [23]. DMG and glutarate are derived from choline and lysine metabolism, respectively; (3) Compounds derived from both microbiota and dietary components (e.g., TMAO). TMAO is produced from the breakdown of choline and l-carnitine, which are metabolized by intestinal bacteria into trimethylamine and then converted to TMAO in the liver.

Of note, the toxicity of some uremic toxins is indirectly potentiated by the intestinal microbiota, as was described for urea. Urea is the major vehicle for the elimination of nitrogen waste arising during the catabolism of amino acids. The production of urea is not directly influenced by intestinal microbiota; However, the microbial enzyme urease issued form the gut microbiota hydrolyzes urea and locally produces excessive amounts of ammonia [15]. Moreover, during CKD, retention of circulating urea results from its heavy influx into the intestinal tract. This latter point was confirmed using germ-free mice that nicely demonstrated that the microbiota, through the generation of ammonia, decreases the intestinal level of urea [21]. Urea and ammonia in the intestinal tract could also influence the gut microbiota composition and URS production [4]. 

Finally, in contrast, other uremic toxins seem to be independent of the intestinal microbiota, such as asymmetric dimethylarginine, symmetrical dimethylarginine, and guanidinosuccinic acid [24,25].

## 4. Role of Food Composition to Decrease Intestinal URS

Recently, large systemic studies revealed that a healthy diet including many fruits and vegetables, fish, legumes, whole grains, and fibers and also a cutting down on red meat, sodium, and refined sugar intake were associated with a decreased risk of incident CKD for participants consuming the highest quartile of vegetable proteins [26] and a lower mortality in CKD patients [27]. In contrast, red meat intake increased the risk for developing end-stage renal disease [28]. A kidney-friendly diet could help to protect renal function from further damage and a dataset seems to indicate that CKD and its related complications are the consequence of the modification of the intestinal microbiota and the production of URS. Unfortunately, despite the fact that the deleterious influence of certain types of diet/nutrients composition on URS generation has been identified, very few studies explored the impact of this specific diet on CKD progression and patient survival.

### 4.1. Sources of Protein: Red Meat and Vegetarian Diet

Clinical and experimental data have demonstrated that dietary l-carnitine from red meat or fish increases TMAO concentrations [9,29,30]. However, the direct link between l-carnitine intake, plasmatic TMAO concentration, and CV or CKD progression still remains unclear and inconsistent between populations. For example, fish ingestion may increase TMAO excretion more than 20 times when compared with alternative diets, but despite this, higher consumption of fish has for a long time been associated with CV protection [31]. A meta-analysis of 13 randomized controlled trials using l-carnitine supplementation after myocardial infarction reported a significant reduction in mortality [32]. Also in a meta-analysis in hemodialysis patients with l-carnitine supplementation, no such mortality benefit or increase was found, although a benefit was observed on dyslipidemia and some inflammation biomarkers [33]. However, in these studies l-carnitine was administered per os or intravenously and therefore parenteral administration could bypass gut microbial conversion of l-carnitine to TMAO and provide protective effects. 

In addition to providing large amounts of choline and l-carnitine, animal-based diets seem to have a greater impact on altering the gut microbiota than a plant-based diet. For example, red meat changes the intestinal microbiota favoring TMA-forming bacteria such as *Clostridia* and *Prevotella*, thus potentiating elevated plasmatic TMAO levels [7]. A “Vegan gut” profile appears to be unique in several characteristics, including a reduced abundance of pathobionts and a greater abundance of symbionts [34]. Modulation of the gut microbiome through diet and pre- and probiotics has the potential to strongly impact metabolic disease such as obesity and diabetes. However, we do not know whether adopting a vegetarian/vegan diet could stably switch subjects to a more beneficial enterotype and confer long-lasting health advantages in CKD. 

Vegetarian diets that have a low lecithin, choline, and l-carnitine content should elicit a lower production of TMAO. Unfortunately, not all studies were conclusive. Koeth et al. demonstrated that omnivorous human subjects produced more TMAO than vegans or vegetarians following ingestion of l-carnitine [7]. However, Obeid et al. failed to show that vegans and lacto-ovo-vegetarians eating many eggs (i.e., resulting in a high choline intake) differ in plasma concentrations of TMAO [35]. A vegetarian diet is rich in betaine and might overcome competitive inhibition of DMG on BHMT and reduce homocysteine levels. However, studies of betaine supplementation in hemodialysis patients have failed to show any benefit [36]. TMAO and DMG production are strongly linked since bacteria may use betaine to produce TMAO and that could explain the non-significant studies. 

In individuals with a normal renal function, a vegetarian diet reduces the urinary excretion of PCS and IS by approximately 60%, which reflects their decreased production [37]. However, in this study, vegetarian patients consumed a diet with significantly higher content of non-digestible fibers suggesting that the prebiotic effect could have modulated URS production rather than the reduction of animal proteins. It should be noted, however, that in maintenance hemodialysis, a vegetarian diet is associated with reduced IS and PCS plasma concentrations [38]. 

Some observational studies, such as the ARIC Study addressing almost 12,000 adults with normal kidney function, have highlighted that there was no significant association between total protein consumption and incident CKD. However there were significantly increased risks of incident CKD for individuals consuming more red meat and a protective effect for participants with vegetable protein intakes [26]. These results are in good agreement with a recent meta-analysis of 7 cohort studies including 15,285 adults with CKD, reporting that healthy dietary patterns were associated with a reduced risk of all-cause mortality in individuals with CKD [27]. Of note, red meat intake was reported to increase the risk of developing end-stage renal disease [28]. 

### 4.2. Pro- and Pre-Biotics 

Prebiotics are indigestible food additives that promote the selective growth of beneficial bacteria subtypes while limiting proliferation of pathogenic bacteria [19]. Before the rise of the concept of prebiotics, an increase in dietary fiber intake was already proposed in CKD patients as an adjunct to dietary protein restriction to reduce urea concentration [39] and was demonstrated to lower mortality in this population [40]. 

Even if it remains difficult to discriminate whether dietary fiber or other nutrients present in the food rich in fiber provide the health benefits, there is strong evidences that suggest that prebiotics decrease intestinal URS through their effects on gut microbiota. Limited studies in non-CKD people have observed that consumption of resistant starch decreased fecal phenol and ammonia [41]. Using a substrate labeled with a stable isotope of tyrosine, it was demonstrated than inulin (a non-digestive soluble fiber) decreased urinary excretion of both labeled and unlabeled p-cresol [42]. Similarly other prebiotics such as lactulose, oligofructose-enriched inulin or arabinoxylan-oligosaccharides (AXOS^®^) were efficient to decrease urinary excretion of p-cresol while increasing *Bifidobacteria* counts [43,44,45]. The prebiotic resveratrol is a natural polyphenolic compound derived from phytoalexin found in grapes and berries. ApoE^–/–^ mice fed with resveratrol exhibited an increased gut *Lactobacillus*, a decreased plasma TMAO levels, and attenuated level of aortic atherosclerosis [46]. 

In CKD rodent models, Younes et al. showed that fermentable carbohydrates decreased urea concentration [47] and we previously reported that AXOS consumption reduced PCS production [48]. Kieffer et al. have demonstrated that high-amylose maize-resistant starch reduce microbial-derived indole and phenol [49]. In predialysis patients, several studies reported that a supplementation with arabic gum or fermentable carbohydrates was able to decrease serum urea concentration and increase nitrogen in stool [50,51,52]. However, in other studies, no effect of arabic gum was observed on IS level in CKD patients [53]. Recently a small placebo-controlled cross-over study investigated the effect of inulin and demonstrated a significant decrease in serum p-cresol/PCS [54]. Conversely, in the same population, Poesen et al. [55] failed to demonstrate any effect of the prebiotic AXOS on microbiota derived URS (IS, PCS) and urea. There was only a significant decrease of serum concentration of TMAO in the prebiotic group. On the contrary, increasing dietary fibers for six weeks in haemodialysis patients significantly reduced PCS and IS production [56]. 

It is worth noting that the composition of the diet should be carefully selected. For example, a specific type of oligosaccharides or dietary fibers caused the reduction of bacteria species, and selectively promoted the growth of *Bacteroides* or *Alloprevotella* bacteria, resulting in an increase of TMAO, whereas a nutritionally balanced diet decrease TMAO concentration [57]. Finally, in most of these clinical trials, the effect on other microbial metabolites was not studied and it will be interesting to have an exhaustive map of the changes in URS produced by the intestine to better understand the outcomes of these studies.

Other strategies to reduce URS production could be probiotic administration, i.e., administration of a living microorganisms that could confer a health benefit for the host. Similar to the prebiotic strategy, the quality of intervention trials investigating this novel CKD therapy is lacking and many remained inconclusive. The choice of the strains of probiotic used is until now mainly empirical and that could explain the discrepancies between studies. In addition, safety issues should be carefully addressed [4]. Indeed, probiotics resulting in high levels of the urease enzyme may increase the generation of deleterious ammonia from urea. Also, persistence of a uremic environment in the gut may not favor the survival of probiotics and this may limit their potential health benefits. In hemodialysis patients, several studies have reported a sharp reduction in fecal p-cresol production while plasma p-cresol only slightly decreased [58]. In the same population, probiotic consumption decreased plasma concentration of IS [59] but only insignificantly decreased indoxyl glucuronide [60]. Unfortunately, all the studies with probiotics did not yield positive results and recently Borges et al. reported that in the CKD population, probiotics failed to reduce IS concentrations [61]. An association of prebiotic and probiotics, referred to as a synbiotics, could also be interesting. A small randomized controlled trial in 37 stage 4 to 5 CKD patients demonstrated that this combination altered gut microbiota composition and reduced plasma concentrations of PCS [62]. 

A clear picture of the intestinal microbiota profile in CKD is needed to better target the beneficial and deleterious bacteria. For instance, some new exciting strategies are emerging using specific mutant bacteria. Delvin et al. have identified an abundant family of microbial tryptophanases within the human gut and demonstrated that altering the abundance of bacteria harboring tryptophanase activity can serve as a means of modulating IS production in vivo [63]. However, this strategy has not been explored in uremic conditions. 

## 5. Role of Dietary Restrictions on URS Production

### 5.1. Low Protein Diet (LPD) and Ketoacids Supplementation 

Another strategy to prevent URS production could be to reduce the dietary intake of the precursor amino acids of URS. Thus, the poorly understood beneficial effects of LPD may be partially explained through such a mechanism. The optimum protein intake for maintaining kidney health in CKD patients has been the subject of intense and continuous debate even if recent reviews and meta-analyses demonstrated the interest of this nutritional strategy in this population [64,65]. Therefore, the current international recommendation for daily dietary protein intake is 0.6 to 0.8 g/kg/day for non-dialysis CKD patients and 1.1 to 1.2 g/kg/day for those on dialysis [65,66]. In non-dialysis CKD patients, we may be further decreased to 0.3 g/kg/day with a supplementation with ketoanalogues (KA). KA are a mixture of alpha-ketoacid analogues of essential amino acids deprived from the amino group which are further converted to their respective amino acids without providing additional nitrogen [67]. The conversion into amino acids in fact consumes nitrogen which is taken off from the urea cycle and therefore reduces urea production, as evidenced by a lower serum and urinary urea in patients undergoing these diets.

The deleterious impact of high protein intake in CKD has been known for decades [68]. Recently, Poesen et al. have investigated the effects of protein intake on the metabolome of healthy individuals and observed that a high-protein diet increased the plasma levels of deleterious intestinal derived-URS (such as PCS and IS) and urea [69]. Consumption of dietary proteins is proportionately related to the production of urea [70] explaining that LPD reduces urea generation [71]. Recently, some evidences regarding the association between LPD and URS are emerging. For instance, LPD was efficient to reduce IS serum levels in CKD rats [72]. In order to reduce even more the protein intake and URS accumulation without nutritional deficiency, a very LPD (22–30 g/day or 0.3 g/kg/day) with KA could be an interesting strategy. Indeed in a prospective randomized controlled trial concerning 32 non-dialysis CKD patients, IS levels were reduced after only 1 week of LPD supplemented with KA even when preceded by a conventional LPD [73]. However, it remains unclear if LPD+KA could also affect the microbiota to decrease URS production or it is only the consequence of reduction of protein intake. A recent pilot prospective study has reported that LPD significantly reduces TMAO plasma concentration [74]. However, no large randomized controlled trials of LPD/LPD + KA has been designed to test the effectiveness of this dietary strategy at reducing URS and are eagerly needed. 

### 5.2. Composition of Amino Acids 

In addition to the quantity of protein intake, research should be done on the impact of the amino acid composition of proteins on URS production and CKD progression. Indeed, regarding the production of TMAO, using a synthetic diet with a choline analogue such as 3,3-dimethyl-1-butanol was associated with a drop in plasma TMAO levels and a decrease in atherosclerosis progression in mice [75]. Also, oral meldonium, a synthetic structural analogue of l-carnitine precursor γ-butyrobetaine, limited elevations in plasma TMAO were observed after a fish-rich diet and increased urinary TMAO excretion by 35% in human subjects [76].

Park et al. reported that rats fed with a low-methionine diet had a four-fold increase in BHMT activity, and further increases were observed when supplemental betaine or choline were added and altogether this could reduce DMG accumulation [77]. Feeding mice with a diet specifically deprived of a selected amino acid (e.g., branched chain amino acids such as leucine, isoleucine, and valine) was sufficient to improve glucose tolerance and body composition to the same extent as a LPD [78]. Therefore, we hypothesize that the selective reduction of amino acids recognized as precursors of URS (such as tyrosine and tryptophan), could exert a beneficial effect similar to that of LPD. There are some preliminary experimental data that observed a decrease of IS in mice fed with low-tryptophan diet [79]. However, in a small cohort of hemodialysis patients, Brito et al. failed to prove an association between tryptophan dietary intake and IS levels. A major weakness of this study was that the tryptophan intake was only parameter evaluated by a 24-h dietary recall performed on 3 different days. This dietary recall could be less accurate than expected and have only provided a “proxy” of tryptophan intake [80]. All these emerging strategies have not been tested in CKD and further studies will be necessary to confirm these nutritional options.

## 6. Conclusions

The current experimental and clinical data available suggest that modulation of the intestinal microbiota could be an interesting target to reduce CKD progression and CV complications by decreasing URS production. Beside diet interventions to reduce URS production by intestinal microbiota, other alternatives should be explored. For example, the intestinal adsorbent AST-120 is always a possibility even if it failed to prevent CKD progression in a large multicentric trial [81]. Large spectra antibiotics is an attractive strategy but cannot be promoted in humans since the expected risks far exceed the foreseeable benefits [82]. The ClC-2 chloride channel activator lubiprostone, a therapy for constipation, favored the recovery of the levels of the *Lactobacillaceae* family and *Prevotella* genus altered in CKD and were able to significantly reduce IS production and progression of kidney disease [83]. Strategies that could decrease all deleterious intestinal microbiota, such as fecal transplantation (which proved very efficient in *Clostridium difficile* infections), could be implemented in CKD patients. 

Finally, it should be noted that the majority of conducted studies were preliminary and more clinical studies needs to be conducted to further understand the effectiveness of these nutritional strategies. In particular, defining a more detailed picture of the intestinal microbiota in CKD to target the pathogenic bacteria and determine the impact of quality composition in addition to the quantity of protein on this dysbiosis are desirable. Instead of a unique nutritional option, a combined strategy which brings together vegetarian diet, LPD, ketoacid supplementation, modulation of quality of protein, and pro/prebiotics could be far more interesting for patients with CKD.
